# The 10–6 Method for Tibial Preparation in Kinematically Aligned Total Knee Arthroplasty: A Technical Description and Preliminary Clinical Evaluation

**DOI:** 10.1016/j.artd.2026.102195

**Published:** 2026-10-29

**Authors:** Philip Mark Anderson, Alexander Nedopil, Moritz Giehl, Axel Jakuscheit, Maximilian Rudert, Jan Oberfeld

**Affiliations:** Department of Orthopedics, Orthopädische Klinik König-Ludwig-Haus, University of Wuerzburg, Würzburg, Germany

**Keywords:** Kinematic alignment, Total knee arthroplasty, Personalized alignment, OR efficiency

## Abstract

**Background:**

Kinematically aligned total knee arthroplasty (KA TKA) aims to restore the proximal tibia's prearthritic varus-valgus (V-V) alignment. However, a standardized thickness to correct for proximal tibia cartilage wear has not been established. This study reports the cartilage thickness of the tibial plateau. Secondary objectives include determining the incidence of V-V recuts when a 4-mm correction for cartilage wear is applied and analyzing the V-V alignment between the preoperative tibial joint line and the postoperative tibial component.

**Methods:**

Using a caliper-based optical method, maximum articular cartilage thickness was measured on 28 resections of the tibial plateaus from 28 patients undergoing unrestricted KA TKA. One surgeon corrected for cartilage wear on the tibial plateau by decreasing the resection thickness by 4 mm using a 10-mm- and 6-mm-stylus (10–6 method). Rates of necessary recuts were documented.

**Results:**

The mean maximum cartilage thickness was 3.8 mm (95% confidence interval 3.4-4.2 mm), with no significant differences by sex or body mass index. The frequency of V-V recuts was 7 %. The difference in V-V orientation of the tibial component from preoperative was 1.2° (95% confidence interval, 1.7°-0.3°)

**Conclusions:**

Because the mean maximum cartilage thickness of the tibial plateau is close to 4 mm, a 4-mm correction by using the 10–6-method results in a low recut rate when performing KA TKA. Surgeons using two styli with a 4-mm difference in resection depth (10–6 method) should expect to accurately restore the prearthritic V-V orientation of the proximal tibial while minimizing the need for recuts.

## Introduction

Since the introduction of modern total knee arthroplasty (TKA) in the 70s of the 20th century, this procedure has undergone tremendous evolution concerning both surgical and manufactural techniques. Despite all the modifications and improvements brought to this field, it was not until the last decade that the concept of “mechanical alignment” (MA) was questioned in an effort to improve the clinical outcomes of TKA and reduce the burden of unsatisfied patients which is as high as 20 % [1], [2]. Thereby, MA aims to implant the femoral and tibial components perpendicular to their respective mechanical axes [3].

As an alternative to MA, kinematically aligned (KA) resurfaces the knee to its pre-arthritic state [4]. While being somewhat revolutionary at the time of its introduction, in the meantime KA has gained widespread acceptance in the orthopaedic community worldwide.

One of the fundamental elements of the KA concept is the hypothesis that the prearthritic cartilage thickness of the distal femur is 2 mm [5]. This presumption allows the surgeon during TKA to define the former femoral joint line and to recreate the prearthritic articular surfaces of the knee, making the femoral preparation during TKA straightforward.

In contrast, the tibial preparation is far more challenging as there is no comparable guidance to determine the former joint line on the tibial side. Usually, a preliminary conservative bone cut is recommended, followed by tibial recuts to balance the knee based on the well-defined femoral surface [6]. Importantly, these recuts are not merely occasional corrections but represent an inherent part of the originally described unrestricted calipered KA technique. However, each additional recut increases operative complexity, prolongs surgical time, and removes additional bone. To date, no simple and widely applicable method has been described to guide the initial varus-valgus (V-V) orientation of the tibial resection based on an anatomical estimate of cartilage loss.

Therefore, the goal of the present study was to establish such a technique. By applying a predefined correction for tibial cartilage wear, it aims to reproduce the prearthritic tibial joint line more accurately during the initial resection and thereby reduce the need for subsequent tibial recuts while preserving the principles of unrestricted calipered kinematic alignment. This so called “10–6-method” is based on the assumption that the worn cartilage on the tibial side was 4-mm thick at its maximum, as indicated by former anatomical studies [7]. In cases with a worn side without a bone defect and an unworn side, the V-V orientation of the tibial resection is therefore defined by two styli: one 10-mm stylus touching the cartilage at the highest point of the unworn side and one 6-mm stylus touching the intact bone at the lowest point of the worn side. The slope and rotational orientation of the tibial resection are set in the usual manner using a resection guide and a line bisecting the lateral plateau, as described before [6], [8], [9]. By doing so, the prearthritic tibial surface is reproduced, with the assumption of a 10-mm insert and a loss of 4-mm cartilage on the worn side. This technique is easy and fast to perform and is without the need of any special instruments.

As the 10–6-method has not been described before, this study was set up to (1) evaluate whether the assumption of 4 mm maximum tibial cartilage thickness is justified and to (2) define the rate of necessary tibial recuts by using this technique. The hypotheses were therefore as follows:

1. The maximum cartilage thickness on the unworn tibial plateau is 4 mm.
2. The rate of necessary tibial recuts is below 10 %.

## Material and methods

After approval by the local ethics committee and registration of the study in the German Clinical Trials Register, consecutive patients of one surgeon meeting the predefined inclusion criteria were prospectively recruited and included in the study:

• Age over 18 years
• KA TKA planned due to end stage osteoarthritis with intact contralateral compartment and intact bony surface on the worn side (additional intraoperative confirmation)
• Informed consent by the patient

Aiming for a power of 80 % and setting the level of significance at 0.05 the pre-hoc sample size analysis using a two-sided confidence interval (CI) concluded that 28 patients had to be recruited when assuming that the difference of the cartilage thickness differed maximum 1 mm to the supposed 4 mm.

A difference of 1 mm from the assumed cartilage thickness of 4 mm was considered clinically relevant, as smaller deviations are difficult to detect and correct reliably during surgery given the thickness of standard oscillating saw blades and the reported accuracy of caliper-based measurements [10].

The demographics of the study population are shown in Table 1.

**Table 1:** Demographics of the study population.

Variable	Mean (range)	N (rate in %)
Age (y)	70 (60-86)	
BMI (kg/m^2^)	32 (23-44)	
Gender		
Women		10 (36)
Men		18 (64)
ASA score		
1		1 (4)
2		17 (61)
3		10 (36)
4		0 (0)
Used implants		
NexGen Cr flex		22 (79)
Persona Cr		2 (7)
GMK sphere		4 (14)
Inlay heights (in mm)		
10		28 (100)
Duration of surgery (in min)	67 (43-90)	
Patella resurfaced		
Yes		9 (32)
No		19 (68)

ASA, American Society of Anesthesiologists; BMI, body mass index; kg, kilogram; m, meter; mm, millimeter; min, minutes; N, number.

All included patients underwent KA TKA by one high-volume arthroplasty surgeon. Femoral preparation was performed in a standard caliper-verified KA technique, whereas tibial preparation was performed using the 10–6 method for V-V orientation of the cutting block as described. The resected tibial plateau was then split using an osteotome at the contact point of the stylus with the unworn articular surface. Digital pictures were taken of the cross-section of the split plateau, including a caliper for later referencing (Fig. 1). The number of necessary tibial recuts to achieve a balanced knee was documented. Recuts were defined as cuts performed in order to change the V-V orientation. Cuts for additional space without changing the orientation of the first cut were not defined as recuts. Balance was defined as a rectangular stable extension space with a maximum 2 mm asymmetry during varus- and valgus-stress and a trapezoidal flexion space with a stable medial side during trialing. No tensioning device or instrumented balancing system was used. Balancing was performed according to the principles of manual unrestricted calipered kinematic alignment. Varus and valgus stress were manually applied in full extension and flexion, and the medial and lateral compartment gapping was assessed by the operating surgeon [11].

**Figure 1:**
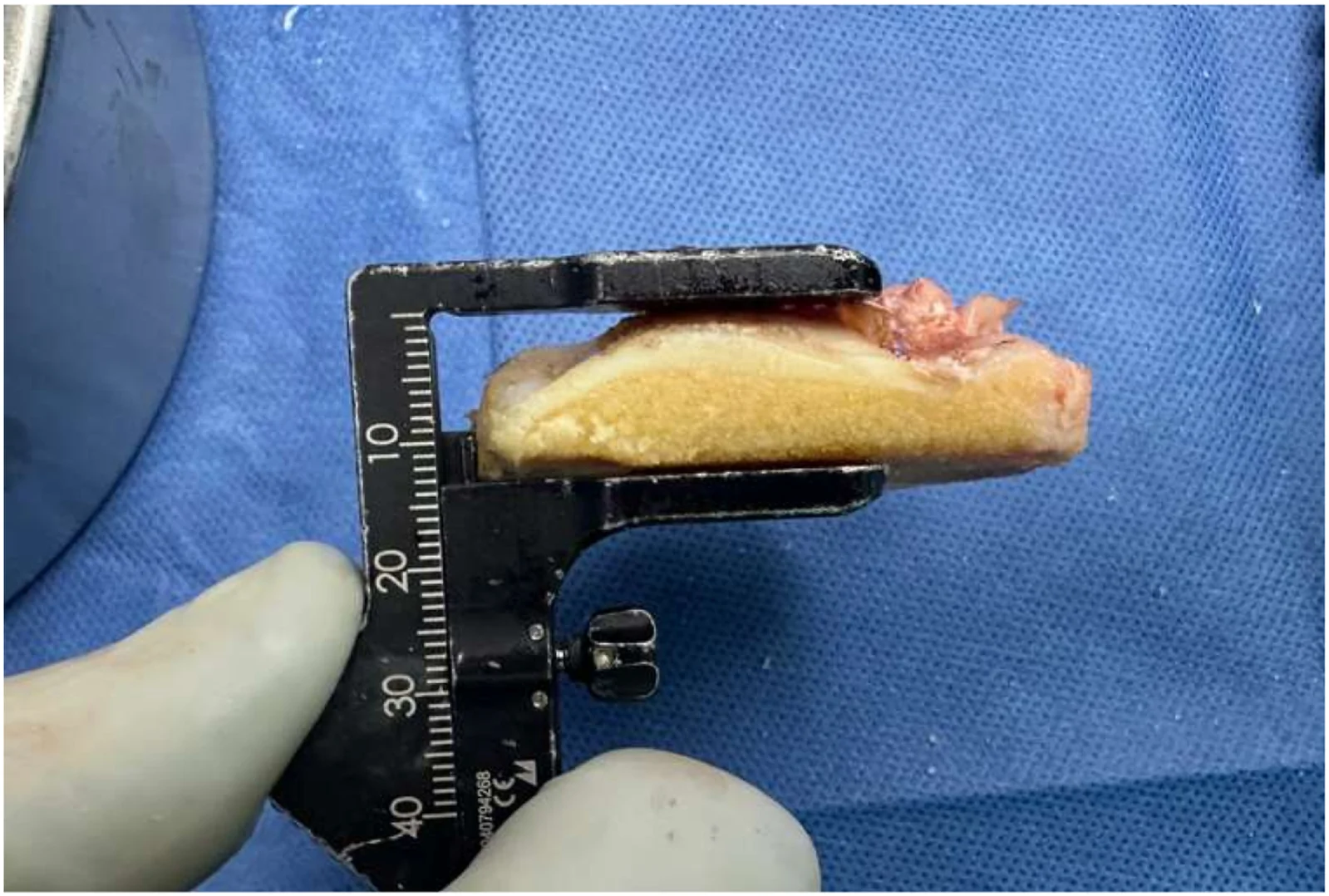
Cross-section of the unworn side of the tibial plateau with a caliper for referencing.

After surgery, the thickness of the cartilage was measured digitally by dedicated software (ImageMeter, version 3.9.4-8; Algorithmic research, Stuttgart, Germany) using the photographed caliper for calibration (Fig. 2) as performed in former studies [10]. The measured values were rounded and are presented as whole numbers without decimals. This approach was chosen because the purpose of the study was to evaluate a surgical technique applicable in routine clinical practice. Intraoperative measurements and corrections of ˂1 mm are difficult to perform reliably and are of limited practical relevance. Reporting decimal values would therefore imply a level of precision that is generally hardly achievable during standard TKA.

**Figure 2:**
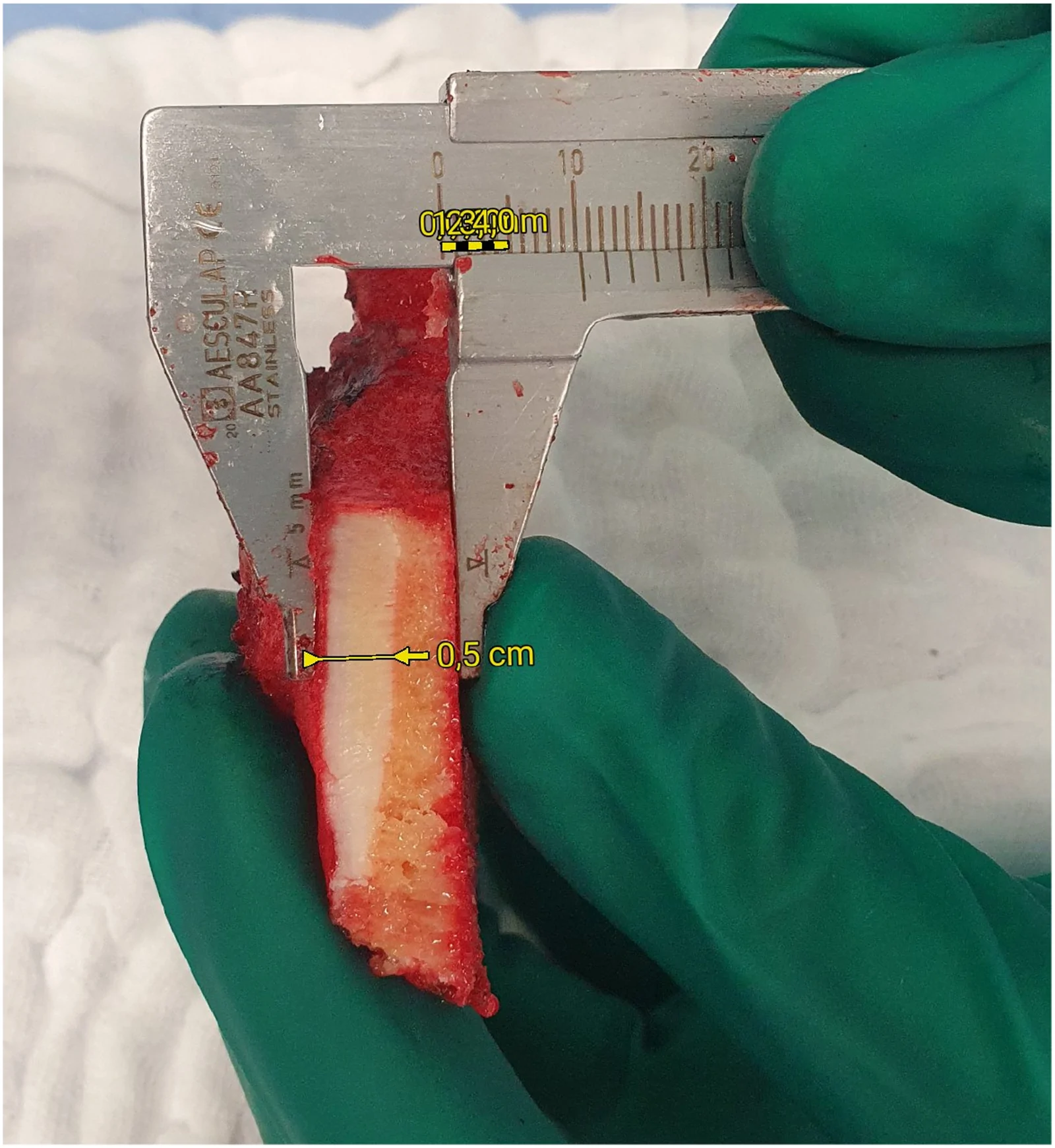
Digital measurement of the cartilage thickness using the caliper as reference.

The preoperative and postoperative medial mechanical proximal tibial angle (MMPTA) was measured twice by two fellowship-trained orthopaedic surgeons on long-leg radiographs (Fig. 3) and the difference computed as a surrogate parameter for reconstructing the prearthritic tibial anatomy (postoperative minus preoperative MMPTA) as intended by KA TKA.

**Figure 3:**
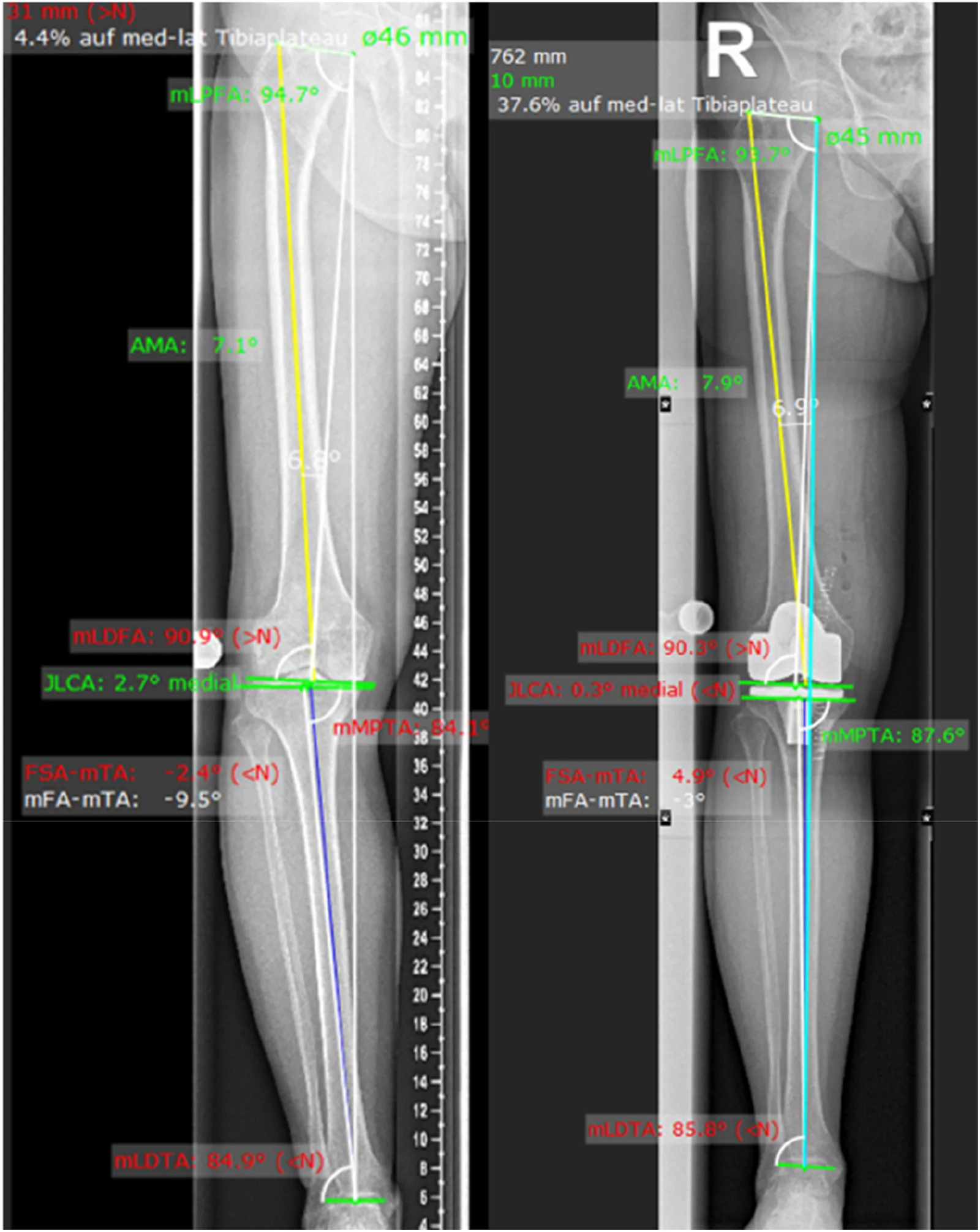
Preoperative and postoperative long leg radiographs. MMPTA and MLDFA were measured. MLDFA, mechanical lateral distal femoral angle.

Additionally, the preoperative and postoperative mechanical distal femoral angles were measured (Fig. 3), and phenotyping via coronal plane alignment of the knee (CPAK) classification was performed [12].

Statistical analysis was performed with SPSS software version 28.0 (SPSS Inc., Chicago, IL). Categorial data are presented as counts and rates. Numerical data are presented as means and ranges.

Comparison of two groups concerning numerical data was conducted via two-sided *t*-test or Mann–Whitney rank-sum test in case of failed normality test (Shapiro–Wilk). Correlations were analyzed by Spearman correlation test.

Intra-rater and inter-rater reliability of the radiographic measurements were assessed using intraclass correlation coefficients (ICCs).

To assess intra-rater reliability, the two repeated measurements performed by each examiner on all radiographs were compared separately. A two-way mixed-effects model with absolute agreement was applied.

For the assessment of inter-rater reliability, the mean value of the two measurements for each examiner was calculated for every radiograph. Agreement between the two examiners was evaluated using a two-way random-effects model with absolute agreement based on mean measurements.

ICC values and CIs are reported. Interpretation of ICC values followed commonly accepted guidelines (<0.50 poor, 0.50-0.75 moderate, 0.75-0.90 good, and >0.90 excellent reliability).

## Results

The mean maximum cartilage thickness was 3.8 mm (95% CI 3.4-4.2 mm). The distribution of cartilage thickness is shown in Figure 4. In 25 patients (89 %) the thickness was within a range of 4 ± 1 mm.

**Figure 4:**
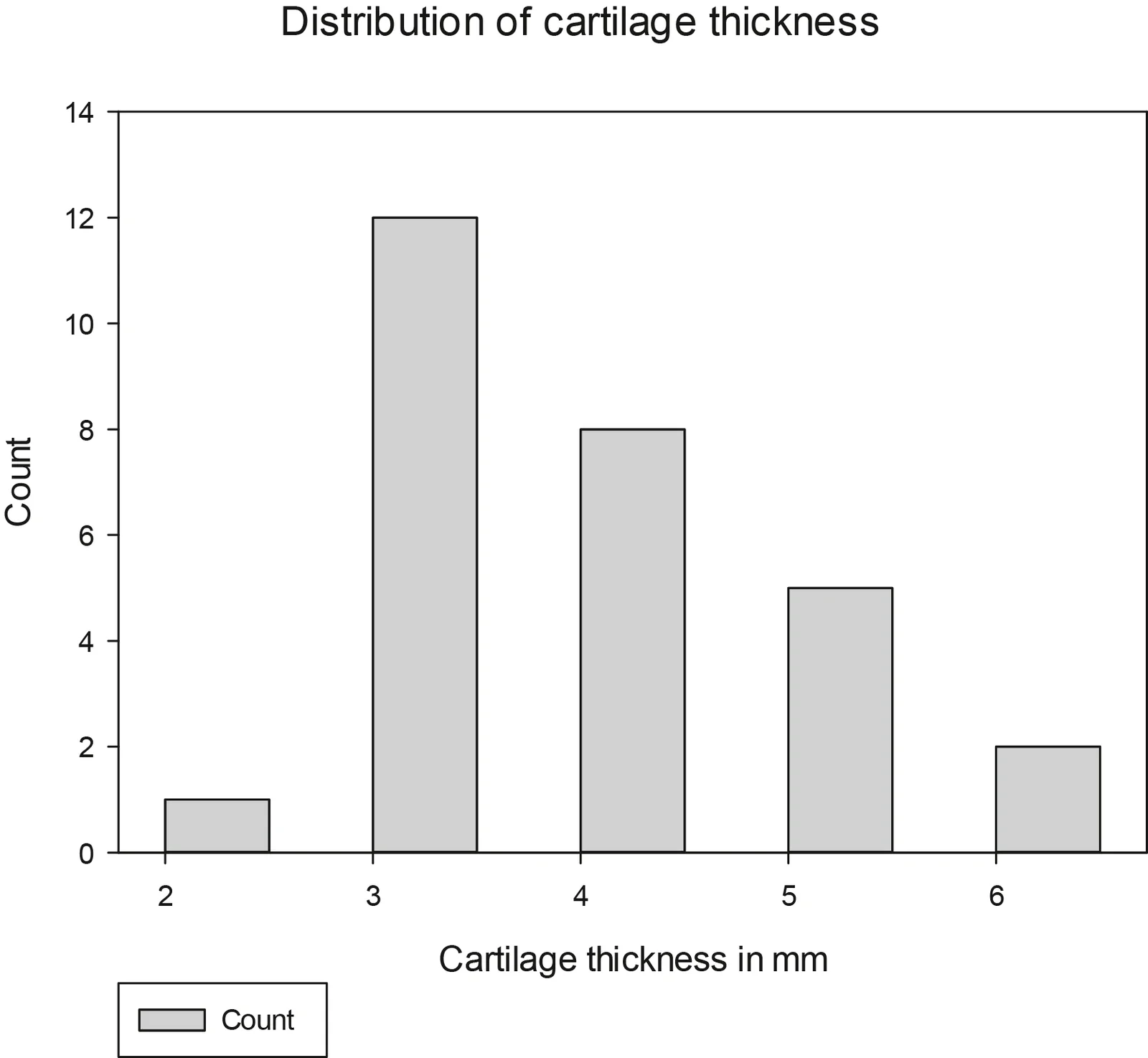
Distribution of cartilage thickness.

In 25 (89 %) patients, the lateral side was unworn, reflecting the more common varus arthritis. The thickness between the lateral (mean 3.8 mm, 95% CI 3.5-4.2) and medial side (mean 3.0 mm, 95% CI 0.5-5.8) did not differ significantly (*P* = .181).

Regarding the sex of the patients there was no significant difference concerning the thickness of the cartilage (*P* = .290). Patient's height, as well as the patient's bone dimensions—mirrored by the implant sizes—were not associated with the measured cartilage thickness (*P* > .05).

Tibial recuts for changing the V-V orientation were necessary in 2 patients (7 %). The corresponding cartilage thicknesses in these two patients were 4 mm and 5 mm, respectively.

Concerning the radiographic measurements, intra-rater reliability was excellent for both examiners with ICC of 0.94 (95% CI: 0.90-0.97) and 0.92 (95% CI: 0.87-0.96), respectively.

Inter-rater reliability between the two examiners was also excellent with an ICC of 0.93 (95% CI: 0.89-0.96).

The mean difference between postoperative and preoperative MMPTA was 1.2° (95% CI, 0.3°-1.7°) (*P* = .128).

The distribution of preoperative and postoperative phenotypes according to the CPAK classification is shown in Figures 5 and 6.

**Figure 5:**
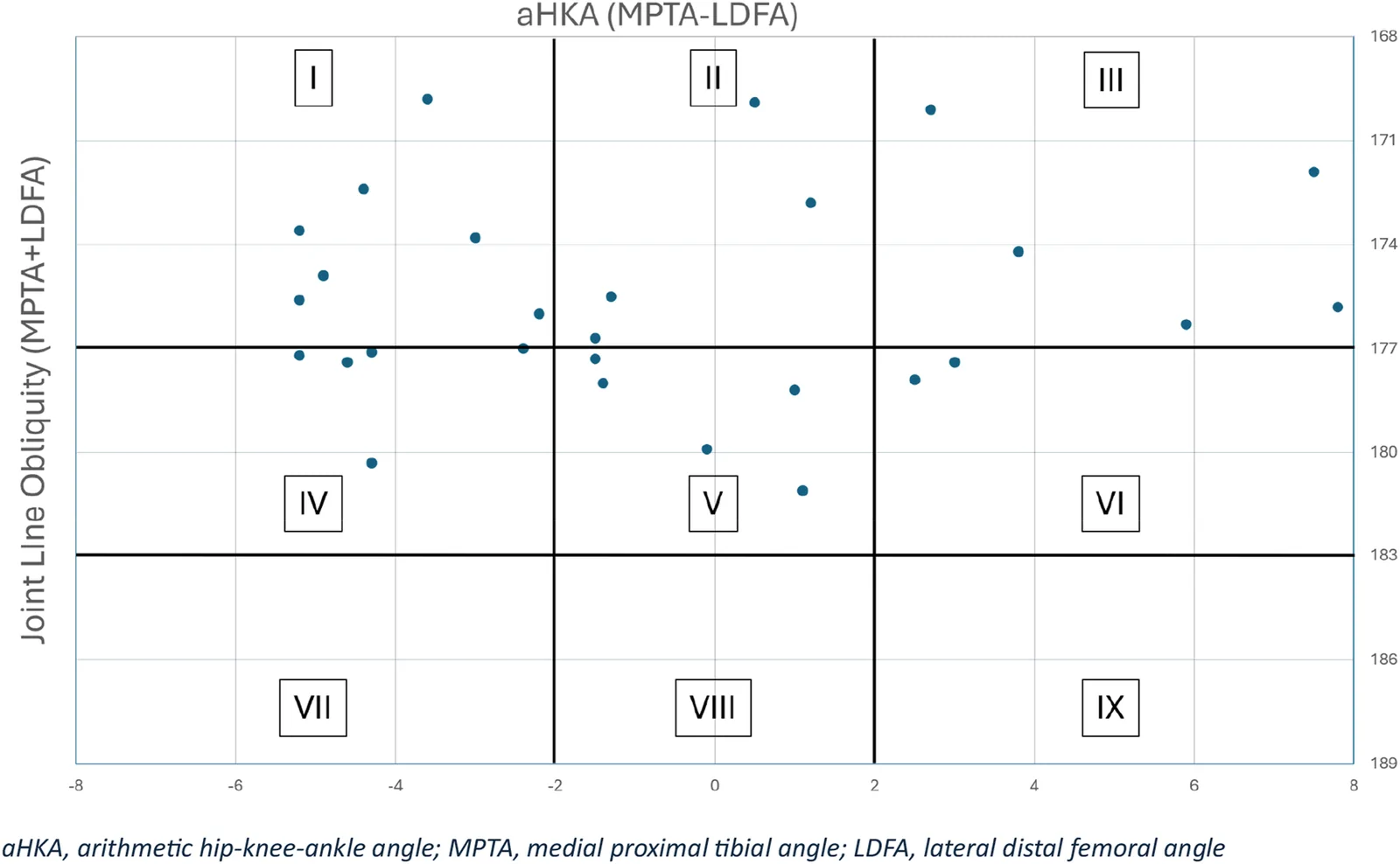
Distribution of preoperative CPAK phenotypes. aHKA, arithmetic hip-knee-ankle angle; LDFA, lateral distal femoral angle.

**Figure 6:**
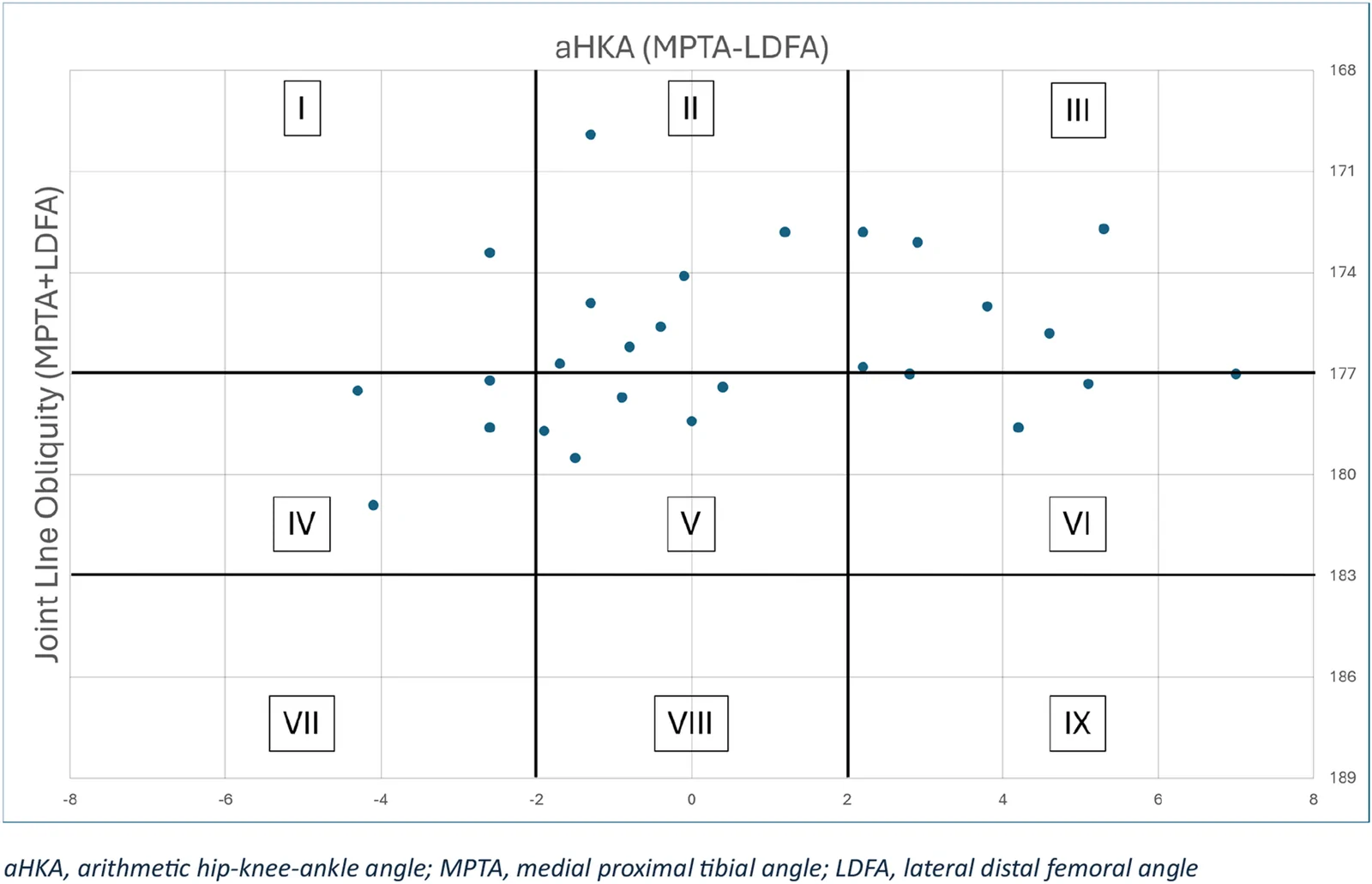
Distribution of postoperative CPAK phenotypes. aHKA, arithmetic hip-knee-ankle angle; LDFA, lateral distal femoral angle.

## Discussion

The two most important findings of the present study were that 1) the mean maximum cartilage thickness of the proximal tibia was 3.8 mm (95% CI 3.4-4.2 mm) and 2) when compensating for 4 mm cartilage wear (ie, the 10–6 method), the frequency of tibial recuts was below 10%.

Other studies before reported on the thickness of the tibial cartilage. Kladny et al [13] measured the thickness in 14 cadavers by histological sections and magnetic resonance imaging (MRI). They found values between 0.9 mm and 6.6 mm, whereby some specimens showed signs of osteoarthritis. Ateshian found the tibial cartilage to be 3.5 mm thick on average on the lateral side, consistent with the previously reported results. In contrast, they found the cartilage on the medial side to be on average 1 mm thinner [7]. Cohen reported an average thickness of the tibial cartilage of 2.4 mm in an MRI study on 6 cadavers. Eckstein in an MRI study reported even lower values of tibial cartilage thickness. They found the medial cartilage to be 1.6 mm on average and the lateral one to be 2.2 mm, respectively. The high variability was pronounced [14]. In another MRI and computed tomography study by Eckstein, the maximum cartilage thickness on the tibia was found to be 4 to 6 mm, better reflecting the results of the present study [15].

In summary, former studies showed a trend to thinner cartilage thicknesses on the tibia than in the present study but most of them used MRI and had small sample sizes. Furthermore, often cadavers were used as specimens. Possible reduction of cartilage thickness by loss of water after death might have occurred. Finally, the cartilage in the presented study was measured at the highest point of the plateau, therefore representing the maximum thickness, whereas the former studies measured the thickness at multiple locations and therefore measured the average thickness.

Even though nearly 90% of patients in our cohort demonstrated a maximum cartilage thickness within ±1 mm of the assumed value of 4 mm, supporting the practical applicability of the proposed correction factor in this patient population, possible variability related to age, sex, ethnicity, body size, and individual anatomy should be acknowledged. The two patients requiring tibial recuts represented opposite ends of the height spectrum, measuring 190 cm (6 ft 3 in) and 150 cm (4 ft 11 in), respectively. While the sample size is too small to infer any association, these cases might highlight the potential influence of individual anatomical variability on cartilage thickness and surgical planning.

The knowledge of a 4-mm thick tibial cartilage at its maximum allows a measured resection technique similar to the femoral side by using two styli, 10 mm and 6 mm, respectively. By doing so, the V-V orientation of the proximal tibia is reconstructed precisely as was shown by the low rate of necessary recuts and the high accordance of the preoperative and postoperative MMPTAs.

Nearly 90 % of the patients showed cartilage thicknesses between 3 mm and 5 mm, making the 10–6 method universally applicable. While larger sample sizes are necessary to reinforce these results, neither sex, patient size correlated with the thickness of the tibial cartilage in this study population making the 10–6 method widely adoptable.

Especially nowadays, where a lot of effort is undertaken to reduce costs in the healthcare system, the 10–6 method is therefore an efficient way to reduce potential time-consuming recuts without adding substantial costs to the procedure.

Next to the saving of time, the tibial bone, too, is preserved to the best possible extent. With all patients the thinnest available inlay was used, underlining this. As rates of revisions are rising, this might be a potential advantage in the future.

Despite these advantages, of course, as with every technique, potential limitations have to be taken into consideration. First, the precision of the cut by a jig-guided oscillating saw is not perfect, especially in the presence of sclerotic bone on the worn side. Deviations up to 3.5° from the intended cut have been reported [16]. Second, there are outliers concerning the cartilage thickness, but they are seldom, as shown. Third, this technique is limited to a tibial plateau with exclusive cartilage wear on one side. When bony wear appears or the cartilage is damaged on both sides, adjustments are necessary, as on the femoral side. These can easily be addressed by changing, for example, to a 4-mm stylus instead of a 6-mm stylus when 2 mm of bone wear is suspected. In cases of worn cartilage on both sides without bone defect, two 6 mm styli can be used instead.

To the best of our knowledge, only one other technique specifically intended to reduce tibial recuts after the initial resection has been described. The “LINK device” has been introduced to adjust the V-V orientation of the tibial cut based on ligament tension after reconstruction of the femur. While this approach may reduce the need for recuts, it relies on the surgeon's assessment of native ligament tension. Especially on the lateral side the tension of the collateral ligament in flexion is highly variable and therefore it might be difficult to determine the right V-V orientation by ligament guidance in flexion.

While there is no officially published data on the rates of necessary recuts by using this device, experience with this surgical technique was presented by Maslaris et al [17] at the annual meeting of the “Personalized Arthroplasty Society” in 2025 in Rome. They reported on 100 consecutive KA TKAs using the LINK device and stated that recuts for V-V-reorientation were necessary in 3.4 %.

While one of the “ten commandments” of KA TKA is not to rely on the first tibial cut, rates of necessary recuts by conventional methods have scarcely been published to date [18]. One of the only reports on recut rates is from Sappey-Marinier in 2023 reporting on the success of robot-assisted KA TKA in reaching full extension in cases of severe flexion contracture. Their recut rates varied between 56 and 43 %, but there was no distinction whether the recuts were necessary for V-V-reorientation or for more space.

For this, comparisons concerning the efficiency of the presented 10–6 method are difficult to make. Nevertheless, the rate of recuts was similar to the reported 3.4 % by the LINK-device and much lower than in the robotic-arm assisted KA TKAs.

When considering the presented results, potential confounders have to be taken into account. The number of patients included was rather small, but this is explained by pre hoc sample size analysis as described. By including more patients, some differences between sexes and knee sizes might become evident as indicated by former studies [5], [7], [14]. Nevertheless, a missed target can be corrected by recuts, so no bridges are being burned by using the 10–6 method.

Additionally, the location of the measurement of the cartilage thickness is debatable. As mentioned, it was measured at the site where the stylus was positioned before resection. The stylus was set at the highest point of the unworn side and the lowest point of the worn side. By changing the site of measurement, the thickness of the cartilage might differ slightly. Furthermore, even though care was taken to split the tibial plateau perpendicular to the resected surface, some obliquity might have occurred possibly introducing minimal measurement error due to the “parallax effect” [19]. Finally, the success of the tibial cut was defined by the surgeon interpreting the extension gap as “balanced” when there was no asymmetric laxity ˃2 mm by V-V stress. Even though this is common practice in KA TKA, there might be inter-rater differences by divergent surgeons when defining the gap as “symmetric.” Nevertheless, the high accordance of the preoperative and postoperative MMPTAs in tibias without bone loss supports the assessment by the surgeon to be correct.

## Conclusions

The present findings suggest that the 10–6 method is a feasible approach for tibial preparation during unrestricted kinematically aligned TKA and may reduce the need for tibial recuts. However, given the single-surgeon design and absence of comparative or clinical outcome data, further studies involving multiple surgeons and institutions are required to confirm the reproducibility, generalizability, and clinical relevance of this technique.

## CRediT authorship contribution statement

**Philip Mark Anderson:** Writing – review & editing, Writing – original draft, Project administration, Formal analysis, Data curation, Conceptualization. **Alexander Nedopil:** Writing – review & editing, Methodology. **Moritz Giehl:** Writing – review & editing, Supervision. **Axel Jakuscheit:** Writing – review & editing, Methodology. **Maximilian Rudert:** Supervision. **Jan Oberfeld:** Writing – review & editing, Validation, Data curation.

## Funding

This publication was supported by the Open Access Publication Fund of the University of Wuerzburg.

## Conflict of interest

Alexander Nedopil receives royalties from Medacta and honoraria for speakers bureau activities or paid presentations for Medacta; is a paid consultant for Medacta and Smith and Nephew; serves on the editorial/governing board for KSSTA; serves as a board or committee member for European Knee Society and Personalized Arthroplasty Society. All other authors declare no potential conflicts of interest.

For full disclosure statements refer to https://doi.org/10.1016/j.artd.2026.102195.

## Appendix A Supplementary data

Conflict of Interest Statement for Anderson

Conflict of Interest Statement for Nedopil

Conflict of Interest Statement for Jakuscheit

Conflict of Interest Statement for Rudert

Conflict of Interest Statement for Giehl

Conflict of Interest Statement for Oberfeld
