# Supplementary material for: The 10–6 Method for Tibial Preparation in Kinematically Aligned Total Knee Arthroplasty: A Technical Description and Preliminary Clinical Evaluation
Source: Arthroplast Today. 2026 Oct 29;42:102195. doi: 10.1016/j.artd.2026.102195 (PMC13742312; doi:10.1016/j.artd.2026.102195)
Supplement: Supplementary file 1 [file mmc2.pdf]

INDIVIDUAL CONFLICT OF INTEREST STATEMENT

American Association of Hip and Knee Surgeons

(Adopted from the American Academy of Orthopaedic Surgeons disclosure statement)

The following form **must be filled out completely and submitted by each author (example, 6 authors, 6 forms). All items require a response. If there is no relevant disclosure for a given item, enter "*None.*"**

The 10-6-method– an efficient technique to facilitate the tibial preparation in kinematic aligned total knee arthroplasty

1. Royalties from a company or supplier (The following conflicts were disclosed)
   Medacta

2. Speakers bureau/paid presentations for a company or supplier (The following conflicts were disclosed)
   Medacta

3A. Paid employee for a company or supplier (The following conflicts were disclosed)
   none

3B. Paid consultant for a company or supplier (The following conflicts were disclosed)
   Medacta, Smith and Nephew

3C. Unpaid consultants for a company or supplier (The following conflicts were disclosed)
   none

4. Stock or stock options in a company or supplier (The following conflicts were disclosed)
   none

5. Research support from a company or supplier as a Principal Investigator (The following conflicts were disclosed)
   none

6. Other financial or material support from a company or supplier (The following conflicts were disclosed)
   none

7. Royalties, financial or material support from publishers (The following conflicts were disclosed)
   none

8. Medical/Orthopaedic publications editorial/governing board (The following conflicts were disclosed)
   KSSTA

9. Board member/committee appointments for a society (The following conflicts were disclosed)
   European Knee Society, Personalized Arthroplasty Society

Each author must sign AND print or type his/her name, date and submit a separate form

In addition, one BLINDED Conflict of Interest form (no author names used) should be submitted per manuscript with all author disclosures.

Alexander Nedopil	[signature]	2/07/26
Author Name (Print or Type)	Author Signature	Date
