# Supplementary material for: The 10–6 Method for Tibial Preparation in Kinematically Aligned Total Knee Arthroplasty: A Technical Description and Preliminary Clinical Evaluation
Source: Arthroplast Today. 2026 Oct 29;42:102195. doi: 10.1016/j.artd.2026.102195 (PMC13742312; doi:10.1016/j.artd.2026.102195)
Supplement: Supplementary file 2 [file mmc3.pdf]

CONFLICT OF INTEREST STATEMENT

The Journal of Arthroplasty

(Adopted from the American Academy of Orthopaedic Surgeons disclosure statement)

The following form **must be filled out completely and submitted by each author (example, 6 authors, 6 forms). If no discloser is required, please write/type "none" at the end of each sentence.**

Manuscript Title The 10-6-method - an efficient technique to facilitate the tibial preparation in kinematically aligned TKA

1. Royalties from a company or supplier (The following conflicts were disclosed)
   /
2. Speakers bureau/paid presentations for a company or supplier (The following conflicts were disclosed)
   /

3A. Paid employee for a company or supplier (The following conflicts were disclosed)
/

3B. Paid consultant for a company or supplier (The following conflicts were disclosed)
/

3C. Unpaid consultants for a company or supplier (The following conflicts were disclosed)
/

4. Stock or stock options in a company or supplier (The following conflicts were disclosed)
   /
5. Research support from a company or supplier as a Principal Investigator (The following conflicts were disclosed)
   /
6. Other financial or material support from a company or supplier (The following conflicts were disclosed)
   /
7. Royalties, financial or material support from publishers (The following conflicts were disclosed)
   /
8. Medical/Orthopaedic publications editorial/governing board (The following conflicts were disclosed)
   /
9. Board member/committee appointments for a society (The following conflicts were disclosed)
   /

Each author must sign AND print or type his/her name, date and submit a separate form.

In addition, one BLINDED Conflict of Interest form (no author names used) should be submitted per manuscript with all author disclosures.

JAKUSCHEIT, AXEL [signature] 2/6/26

Author Name (Print or Type) Author Signature Date
