# Supplementary material for: The 10–6 Method for Tibial Preparation in Kinematically Aligned Total Knee Arthroplasty: A Technical Description and Preliminary Clinical Evaluation
Source: Arthroplast Today. 2026 Oct 29;42:102195. doi: 10.1016/j.artd.2026.102195 (PMC13742312; doi:10.1016/j.artd.2026.102195)
Supplement: Supplementary file 5 [file mmc6.pdf]

CONFLICT OF INTEREST STATEMENT

The Journal of Arthroplasty

(Adopted from the American Academy of Orthopaedic Surgeons disclosure statement)

The following form **must be filled out completely and submitted by each author (example, 6 authors, 6 forms). If no discloser is required, please write/type "none" at the end of each sentence**.

Manuscript Title The 10-6-method - an efficient technique to facilitate the tibial preparation in kinematically aligned TKA

1. Royalties from a company or supplier (The following conflicts were disclosed)

2. Speakers bureau/paid presentations for a company or supplier (The following conflicts were disclosed)

3A. Paid employee for a company or supplier (The following conflicts were disclosed)

3B. Paid consultant for a company or supplier (The following conflicts were disclosed)

3C. Unpaid consultants for a company or supplier (The following conflicts were disclosed)

4. Stock or stock options in a company or supplier (The following conflicts were disclosed)

5. Research support from a company or supplier as a Principal Investigator (The following conflicts were disclosed)

6. Other financial or material support from a company or supplier (The following conflicts were disclosed)

7. Royalties, financial or material support from publishers (The following conflicts were disclosed)

8. Medical/Orthopaedic publications editorial/governing board (The following conflicts were disclosed)

9. Board member/committee appointments for a society (The following conflicts were disclosed)

Each author must sign AND print or type his/her name, date and submit a separate form.

In addition, one BLINDED Conflict of Interest form (no author names used) should be submitted per manuscript with all author disclosures.

Jan Oberfeld	Oberfeld	05.02.2026
Author Name (Print or Type)	Author Signature	Date
